# Non-intubated Video-Assisted Thoracoscopic Pleurodesis Under Paravertebral Block in a Patient With Fibrotic Hypersensitivity Pneumonitis and Chronic Respiratory Failure

**DOI:** 10.7759/cureus.116180

**Published:** 2026-09-13

**Authors:** Afonso Borges de Castro, Rita Tavares de Pina, Miguel Ferreira, Raquel Ferreira Cabral

**Affiliations:** 1 Anesthesiology, Unidade Local de Saúde Estuário do Tejo, Lisbon, PRT; 2 Anesthesiology, Unidade Local de Saúde Santa Maria, Lisbon, PRT

**Keywords:** dexmedetomidine, fibrotic hypersensitivity pneumonitis, non-intubated video-assisted thoracoscopic surgery, paravertebral block, pleurodesis, pneumothorax

## Abstract

Non-intubated video-assisted thoracoscopic surgery (NIVATS) has increasingly been reported as an alternative to general anesthesia in patients at high risk of complications associated with mechanical ventilation. Fibrotic hypersensitivity pneumonitis (FHP) with chronic respiratory failure may pose an indication for this approach, given the risk of positive-pressure ventilation precipitating acute exacerbation of the underlying interstitial lung disease, even in patients with an otherwise uncomplicated intraoperative course.

We report the anesthetic management of a 42-year-old woman with FHP and chronic respiratory failure on long-term home oxygen therapy, who developed a spontaneous pneumothorax refractory to conservative treatment and an initial surgical attempt via video-assisted thoracoscopic surgery (VATS) under general anesthesia. Due to persistent and worsening pneumothorax and given the potential risks of general anesthesia in this setting, she underwent NIVATS pleurodesis two months later under an ipsilateral multilevel thoracic paravertebral block (T2-T7) combined with dexmedetomidine-ketamine sedoanalgesia. This case demonstrates the feasibility of NIVATS pleurodesis under regional anesthesia, combining a multilevel thoracic paravertebral block with sedoanalgesia using agents with limited respiratory depressant effects in a frail patient with fibrotic interstitial lung disease and recurrent pneumothorax.

## Introduction

Hypersensitivity pneumonitis (HP) is an immune-mediated interstitial lung disease caused by repeated inhalation of a variety of organic antigens in susceptible individuals. It is a rare condition, with a prevalence ranging from 1.67 to 2.71 per 100,000 population [1]. HP may present with a fibrotic phenotype, which is associated with a poorer prognosis and increased morbidity and mortality, as well as an increased risk of complications such as pneumothorax [2].

Recurrent spontaneous pneumothorax is a well-established indication for video-assisted thoracoscopic surgery (VATS) with pleurodesis, conventionally performed under general anesthesia with single-lung ventilation via a double-lumen tube or bronchial blocker [3]. In patients with underlying fibrotic interstitial lung disease and chronic respiratory failure, general anesthesia and positive-pressure ventilation carry substantial additional risk as follows: limited respiratory reserve and reduced pulmonary compliance increase the risk of barotrauma, volutrauma, and biotrauma, while one-lung ventilation may be poorly tolerated [4].

Non-intubated video-assisted thoracoscopic surgery (NIVATS) has emerged as an alternative approach that may avoid some of the physiological consequences associated with mechanical ventilation. Spontaneous ventilation, preservation of diaphragmatic motion, and hypoxic pulmonary vasoconstriction support a more physiological ventilation-perfusion relationship. Its feasibility and safety have been increasingly reported in thoracic surgery using regional or local anesthesia combined with sedation [5].

Thoracic paravertebral anesthetic block combined with light-to-moderate sedation has been successfully used as the primary anesthetic technique for non-intubated thoracic surgery, including patients with severe cardiopulmonary compromise [6,7]. However, NIVATS is not suitable for all patients or procedures and requires careful patient selection, adequate analgesia and sedation, close surgeon-anesthesiologist coordination, and readiness for conversion to general anesthesia when necessary.

The use of NIVATS pleurodesis specifically in patients with fibrotic hypersensitivity pneumonitis (FHP) has been rarely described. This case highlights the anesthetic rationale and technical considerations relevant to this patient population, whose severe restrictive respiratory impairment and chronic respiratory failure may increase the physiological challenges associated with general anesthesia and mechanical ventilation.

## Case presentation

We present a 42-year-old woman with FHP and chronic respiratory failure requiring long-term home oxygen therapy at 1 L/min, who was on the waiting list for lung transplantation. She weighed 55 kg, with a BMI of 22.9 kg/m², and was a non-smoker with a history of exposure to four pet birds more than 10 years previously. Baseline SpO₂ was 94% while receiving oxygen at 1 L/min, with a respiratory rate of 15 breaths/min and a functional capacity of approximately 4 metabolic equivalents. Transthoracic echocardiography showed globally preserved left ventricular systolic function and a low probability of pulmonary hypertension, although the main pulmonary artery and its major branches were dilated.

She was admitted to the hospital with a right-sided spontaneous pneumothorax refractory to conservative management and underwent surgical debridement via VATS under general anesthesia. Despite the initial procedure, the right apical pneumothorax persisted and subsequently worsened, leading to the indication for VATS talc pleurodesis approximately two months later. Although the previous anesthetic course was uncomplicated, the patient's severe underlying respiratory impairment and chronic respiratory failure made avoidance of further exposure to tracheal intubation and controlled mechanical ventilation a relevant consideration when planning the subsequent procedure.

Preoperative thoracic computed tomography (CT) revealed a residual pneumothorax with adhesions in the right apex, together with a three-density pattern consistent with FHP (Figure 1). Baseline arterial blood gas analysis on room air showed a partial pressure of oxygen (PaO₂) of 41 mmHg, improving to 62.9 mmHg with oxygen supplementation at 1 L/min and a partial pressure of carbon dioxide (PaCO₂) of 57 mmHg, with a pH of 7.47 and bicarbonate (HCO₃⁻) of 29.5 mmol/L. Respiratory function testing showed a forced expiratory volume in one second (FEV₁) of 0.72 L (25% predicted), a forced vital capacity (FVC) of 0.82 L (23% predicted) with a preserved, slightly increased FEV₁/FVC ratio of 88% (108% predicted), consistent with a severe restrictive pattern. Diffusing capacity for carbon monoxide could not be assessed due to the patient's clinical instability.

**Figure 1:**
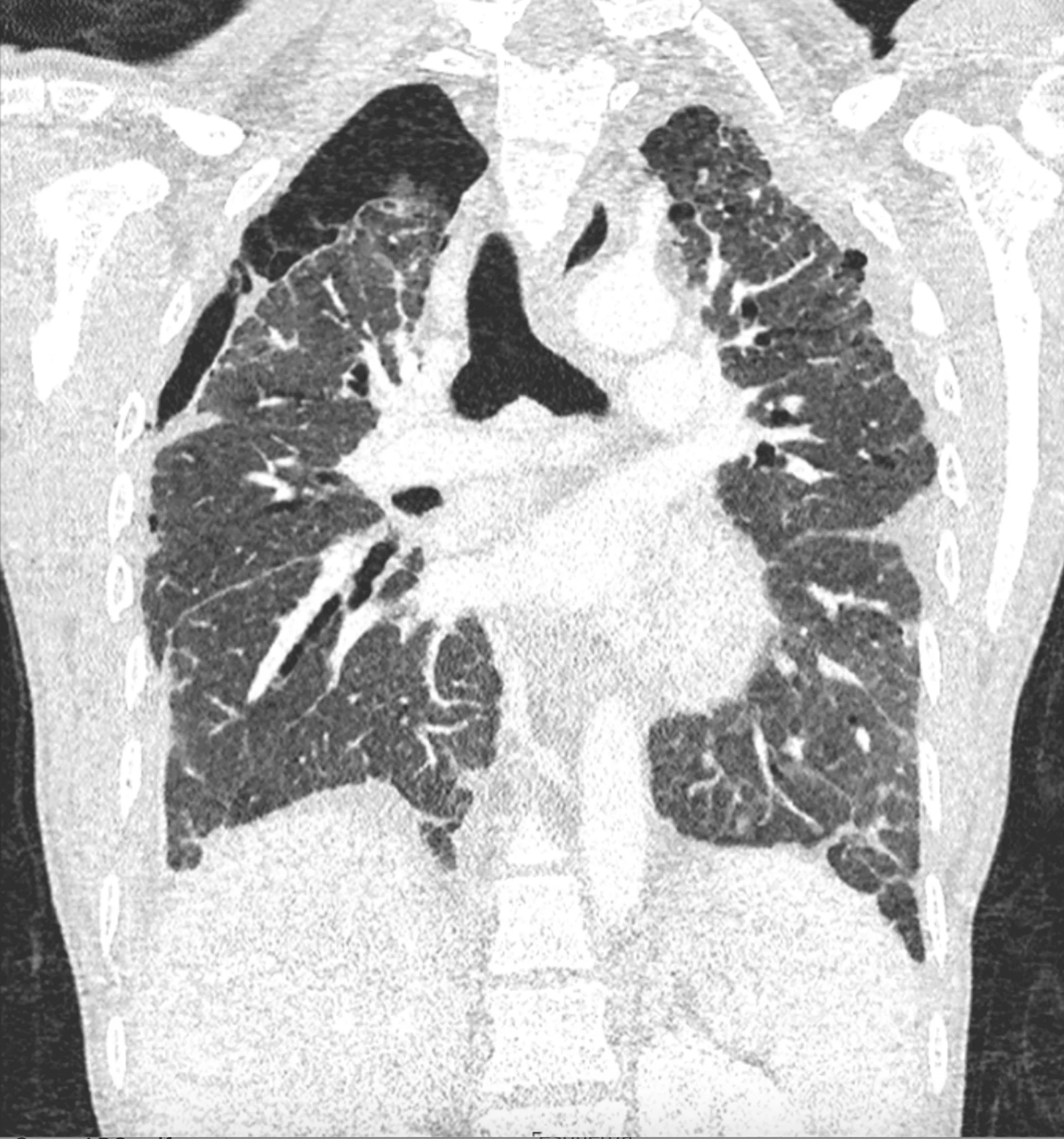
Preoperative thoracic CT. Residual right apical pneumothorax with pleural adhesions and a three-density pattern consistent with fibrotic hypersensitivity pneumonitis.

Preoperative risk assessment

Given her severe underlying restrictive lung disease, chronic respiratory failure, long-term oxygen dependence, and limited pulmonary reserve, general anesthesia with single-lung ventilation was considered to carry substantial physiological risk. A multidisciplinary decision involving the thoracic surgery and anesthesiology teams was therefore made to proceed with a NIVATS approach. The decision was supported by both patient- and procedure-related factors. From a surgical perspective, talc pleurodesis represented a relatively low-complexity thoracoscopic procedure and did not require a completely motionless operative field. From a patient perspective, there was no anticipated difficult airway, significant pulmonary hypertension, persistent cough or copious airway secretions, extensive pleural adhesions, or cardiovascular instability that would preclude a non-intubated approach. Although the patient had severe underlying respiratory disease and limited pulmonary reserve, her respiratory status was clinically stable with supplemental oxygen. These factors were considered favorable for attempting NIVATS.

The patient was positioned in a semilateral decubitus position, allowing adequate surgical access while facilitating airway access and potential tracheal intubation in the event of emergency conversion to general anesthesia. A rescue strategy was planned before the procedure. In the event of severe or refractory hypoxemia, worsening hypercapnia, hemodynamic instability, uncontrolled bleeding, aspiration, or airway injury, the procedure would be interrupted and conversion to general anesthesia performed. The rescue sequence included pausing surgical stimulation, optimizing oxygen delivery and sedation, assisted ventilation when necessary, and progression to tracheal intubation if adequate oxygenation or ventilation could not be maintained. The necessary airway equipment and personnel for emergency airway management were immediately available throughout the procedure.

Preoperative risk assessment classified her as American Society of Anesthesiologists (ASA) Physical Status III [8], with an Assess Respiratory Risk in Surgical Patients in Catalonia (ARISCAT) score of 48, placing her in the high-risk category for in-hospital postoperative pulmonary complications [9]. Table 1 presents the individual components contributing to the score. This risk category is associated with a reported event rate of 42.1%. However, the ARISCAT score does not fully capture the additional pulmonary risk associated with her severe underlying interstitial lung disease and chronic respiratory failure.

**Table 1:** Individual components of the ARISCAT score. ARISCAT: Assess Respiratory Risk in Surgical Patients in Catalonia

ARISCAT risk factor	Patient data	Points
Age	42 years	0
Preoperative SpO₂	≤90% on room air	24
Respiratory infection <4 weeks	No	0
Preoperative anemia	No	0
Surgical site	Intrathoracic surgery	24
Duration of surgery	<2 h	0
Emergency surgery	No	0
Total ARISCAT score	-	48 points

Anesthetic and surgical technique

Regional anesthesia was achieved using an ultrasound-guided multilevel single-shot thoracic paravertebral block performed between the T2-T3, T3-T4, T4-T5, T5-T6, and T6-T7 interspaces, with 5 mL of 0.5% levobupivacaine administered at each level, corresponding to a total dose of 125 mg (approximately 2.3 mg/kg). Fentanyl 50 µg was administered intravenously before performing the paravertebral block.

Intravenous sedation was provided with dexmedetomidine and ketamine, without loading doses, at maximum infusion rates of 0.5 µg/kg/h and 1 mg/kg/h, respectively, titrated to a Richmond Agitation-Sedation Scale (RASS) score of -1 to -2 [10]. Additional intravenous medications included dexamethasone 8 mg, ketorolac 10 mg, and paracetamol 1 g.

High-flow nasal oxygen (HFNO) was administered throughout the procedure at a flow rate of 40 L/min with an FiO₂ of 50%, targeting an SpO₂ >94%, which remained above this target throughout the procedure. Respiratory status was continuously assessed clinically, including respiratory rate, respiratory pattern, chest movement, and work of breathing. Arterial blood gas analysis was performed before and at the end of the procedure to assess oxygenation and ventilation. At the end of the procedure, while receiving HFNO at the same flow rate and FiO₂, arterial blood gas analysis showed a PaO₂ of 95.6 mmHg and a PaCO₂ of 51 mmHg. No clinically significant hypoxemia or respiratory deterioration was observed during the procedure.

The patient remained hemodynamically stable throughout surgery, with no clinically significant deviations from baseline and no requirement for vasopressor support. Crystalloid fluid therapy was administered according to a goal-directed fluid management strategy.

The procedure was uneventful. VATS talc pleurodesis was performed using two thoracoscopic ports, with a chest tube inserted through the fifth intercostal space. Lung collapse caused by the surgical pneumothorax was well tolerated, and pleurodesis was completed under regional anesthesia and sedation without requiring conversion to general anesthesia.

Postoperative course and outcome

She was admitted to a level II intensive care unit (ICU) immediately after surgery as part of the planned postoperative management. She remained hemodynamically stable without respiratory deterioration and was successfully weaned from HFNO during the first postoperative hours, returning to her baseline oxygen requirement of 1 L/min. During the first 24 h, pain scores reached a maximum of 3/10 on the Numeric Pain Rating Scale and were adequately controlled with the paravertebral block and systemic analgesia (paracetamol 1 g and metamizole 1 g every 8 h) [11]. Rescue analgesia with tramadol 100 mg was required only on postoperative day two. A repeat thoracic CT performed on postoperative day four demonstrated resolution of the pneumothorax, and the chest tube was subsequently removed (Figure 2). She was transferred from the ICU to the ward on postoperative day four, where she remained clinically stable while awaiting availability of a bed in a specialized rehabilitation unit. No postoperative complications were documented.

**Figure 2:**
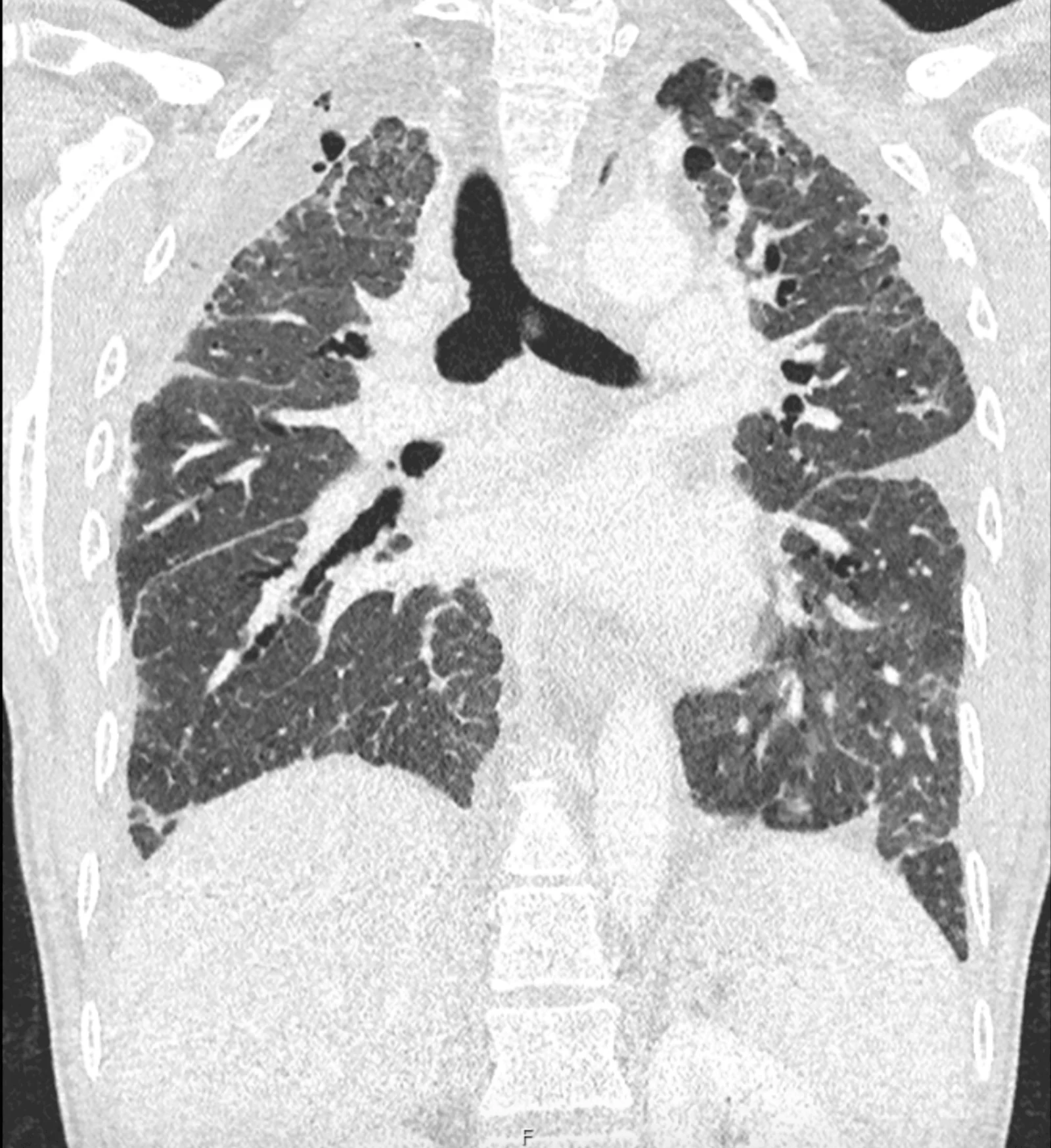
Postoperative thoracic CT. Resolution of the right-sided pneumothorax following talc pleurodesis.

## Discussion

This case illustrates the feasibility of NIVATS talc pleurodesis under combined multilevel thoracic paravertebral block and dexmedetomidine-ketamine sedation in a patient with FHP and chronic respiratory failure. Given her severely impaired pulmonary reserve, avoidance of general anesthesia, endotracheal intubation, and one-lung ventilation was considered desirable. A regional anesthesia-based approach allowed the procedure to be completed while preserving spontaneous ventilation.

NIVATS has been reported across a range of pleural and parenchymal procedures, including pleural biopsy, empyema drainage, and talc pleurodesis for malignant effusions [12-14]. Its use has also been described in patients with severe cardiopulmonary dysfunction, including those with cardiopulmonary failure, using regional anesthesia techniques such as paravertebral block [6,7]. More recently, non-intubated VATS has also been investigated specifically in patients with interstitial lung disease, with observational data suggesting preserved postoperative respiratory function and acceptable perioperative outcomes compared with conventional intubated VATS [15].

Restrictive fibrotic disease raises particular concerns regarding the potential for ventilator-induced lung injury and acute exacerbation following mechanical ventilation, making avoidance of intubation clinically relevant [16,17]. However, the use of NIVATS specifically to avoid these risks in patients with fibrotic lung disease has rarely been reported [15].

In this case, the combination of dexmedetomidine and ketamine sedation offered specific advantages as follows: dexmedetomidine provides sedation and analgesia with relatively limited respiratory-depressant effects, while ketamine contributes analgesia and hemodynamic stability without significant additional respiratory compromise, allowing spontaneous ventilation to be preserved throughout. High-flow nasal oxygen further supported oxygenation during the surgical pneumothorax [18,19], and the multilevel thoracic paravertebral block provided effective segmental anesthesia and analgesia [6,20].

Regarding the choice of regional anesthesia technique, a multilevel thoracic paravertebral block was selected to provide adequate unilateral surgical anesthesia while minimizing hemodynamic effects and facilitating the preservation of spontaneous ventilation. Other options included thoracic epidural anesthesia, which provides dense bilateral thoracic anesthesia but may cause sympathetic blockade and hemodynamic instability, and erector spinae plane block, a simpler and more superficial technique, but with potentially less predictable spread and reliability for surgical anesthesia.

Nevertheless, NIVATS has important limitations and potential complications. Spontaneous ventilation may result in intraoperative hypoxemia or hypercapnia, particularly in patients with limited pulmonary reserve. Respiratory movement, coughing, or inadequate analgesia and sedation may interfere with the surgical field and compromise procedural conditions. Emergency conversion to general anesthesia and tracheal intubation may also be technically challenging, particularly after positioning for thoracic surgery. NIVATS therefore requires careful patient and procedure selection, close coordination between the surgical and anesthesiology teams, continuous monitoring of respiratory and hemodynamic status, and immediate availability of appropriate airway management equipment and personnel in case of emergency conversion to general anesthesia. This case adds to the growing body of evidence describing the use of NIVATS in carefully selected patients with advanced interstitial lung disease, severely compromised pulmonary reserve, and recurrent pneumothorax, particularly when mechanical ventilation is considered high risk.

## Conclusions

NIVATS pleurodesis performed under multilevel thoracic paravertebral block, dexmedetomidine-ketamine sedation, and high-flow nasal oxygen was feasible and well tolerated in this patient with FHP and chronic respiratory failure, with no intraoperative complications. This approach allowed preservation of spontaneous ventilation and avoidance of mechanical ventilation and its associated risks in a patient with severely compromised pulmonary reserve. NIVATS may represent an alternative to general anesthesia in carefully selected patients with advanced interstitial lung disease and recurrent pneumothorax. Nevertheless, given the single-patient nature of this report, further clinical experience is needed to establish its safety and broader applicability in this high-risk population.
